# The inverse relationship between Life’s Essential 8 and risk of metabolic syndrome: evidence from NHANES 2005-2018

**DOI:** 10.3389/fendo.2024.1449930

**Published:** 2024-10-28

**Authors:** Yuhang Liu, Jialing Tang, Siyao Gao

**Affiliations:** ^1^ School of Physical Education and Sports, Central China Normal University, Wuhan, China; ^2^ Department of Physical Education, Central South University, Changsha, China

**Keywords:** Life’s Essential 8, cardiovascular health, metabolic syndrome, NHANES, cross-sectional study

## Abstract

**Background:**

Metabolic syndrome (MetS) has a close association with cardiovascular diseases. Few studies have investigated the association of Life’s Essential 8 (LE8), the updated measurement of cardiovascular health (CVH), with MetS.

**Methods:**

The National Health and Nutrition Examination Survey (2005–2018) data was extracted. The LE8 comprised 4 health behaviors (diet, physical activity, nicotine exposure, and sleep health) and 4 health factors [body mass index (BMI), blood lipids, blood glucose, and blood pressure (BP)]. The total LE8 score is the average of 8 metric scores (0-100), categorized into low (0–49), moderate (50–79), and high CVH (80–100) levels. Multivariable logistic regression models, restricted cubic spline models and stratified analyses were performed to examine the relationship between LE8 and MetS.

**Results:**

In this study, a total of 21,543 participants represented 146.6 million non-institutionalized U.S. adults. Following adjustment for various potential covariates, participants who attained a moderate [adjusted odds ratio (AOR) = 0.234, 95% CI: 0.209, 0.262] or a high CVH level (AOR = 0.026, 95% CI: 0.021, 0.032) exhibited an inverse correlation with MetS risks when comparing those with a low CVH level. An inverse linear dose-response relationship between LE8 scores and MetS risks was also identified (*P* for nonlinearity > 0.05).

**Conclusions:**

LE8 was inversely associated with the risk of MetS. Adhering to LE8 guidelines to sustain a higher CVH level may be beneficial for preventing MetS.

## 1 Introduction

Metabolic syndrome (MetS) represents a group of multiple cardiometabolic factors that include central obesity, hypertension, hyperglycemia, and dyslipidemia (1). The incidence of MetS has risen to epidemic levels, with an estimated 1.5 billion people worldwide yearly (2). About one-third of the U.S. population is affected by MetS (3). The MetS exhibits a close association with an increased risk for cardiovascular disease (CVD), nonalcoholic fatty liver disease, and diabetes mellitus (DM) (1, 4). Additionally, other comorbid conditions of MetS have been increasingly recognized, such as cancer and cognitive degenerative disease (5, 6). Individuals with MetS had a twofold increased risk of developing CVD (7), representing the main mortality cause worldwide (8). Therefore, it is necessary to prevent MetS to minimize the adverse impacts on individual health and medical burden.

In 2022, the American Heart Association (AHA) proposed the Life’s Essential 8 (LE8) as a measurement to assess cardiovascular health (CVH) (9). The LE8 comprised 4 health behaviors [diet, physical activity (PA), sleep and smoking] and 4 health factors [body mass index (BMI), blood lipids, blood pressure (BP), and blood glucose] (9, 10). Since the introduction of LE8, it has spurred research interest. Several studies have found that LE8 was significantly associated with reduced all-cause mortality, cardiovascular mortality as well as a lower risk of multiple chronic diseases (11, 12). Furthermore, Yang and his colleagues discussed the correlation between LE8 scores and metabolic unhealth (MUH) and demonstrated that MUH can be considered as an alternative indicator for LE8 (13). However, little is known concerning the relationship between LE8 and MetS. Given the tight links between the components of LE8 and MetS, improving LE8-evaluated CVH levels may be an appropriate prevention strategy for reducing the burden of MetS.

Despite previous studies exploring the association between CVH and MetS, several limitations are below. First, prior studies included a limited sample size and focused on specific populations. For example, one study only included 517 Atahualpa residents aged ≥ 40 years (14), which confined generalizing the findings to the general population. Second, alcohol consumption and energy intake impact on MetS have been demonstrated (15, 16). However, to our knowledge, only a few researchers have considered these factors, which restricts the capacity to compare and apply their findings to other situations. Third, a majority of prior studies used Life’s Simple 7 (LS7) to assess CVH. Nevertheless, compared with LE8, the LS7-evaluated CVH levels were less sensitive to interindividual differences as well as intraindividual change (9). To compensate for these limitations, based on the National Health and Nutrition Examination Surveys (NHANES) data, we aimed to explore the relationship of the CVH using LE8 scores and MetS in a nationally representative U.S. adult population.

## 2 Methods

### 2.1 Study population

The NHANES is a continuous cross-sectional program conducted by the National Centers for Disease Control and Prevention. Its purpose is to examine the nutritional status, healthy behaviors, and PA outcomes of the non-institutionalized U.S. civilian population (17). The U.S. National Center for Health Statistics' Ethics Review Board granted approval for all NHANES protocols, with all participants signing informed consent to participate in the survey (18).

**Figure 1:**
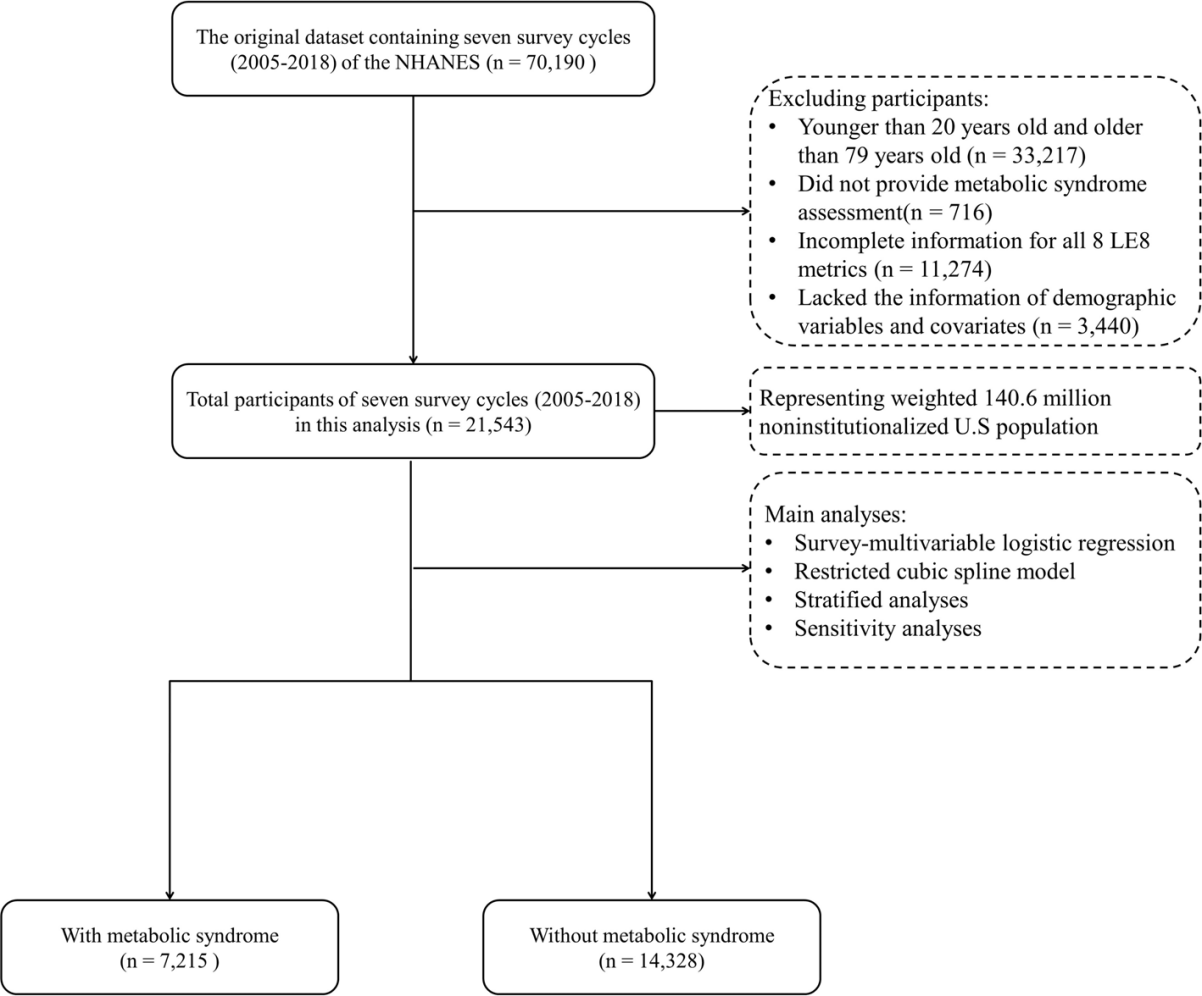
Flowchart of selection of participants.

Herein, we deployed data from several NHANES cycles (2005–2018). First, we excluded participants younger than 20 years and older than 79 years (N = 33,217). Then, this analysis excluded the necessary unavailable covariates (N = 3,440). Participants who did not undergo MetS evaluation (N = 716) and those with inadequate information for all 8 LE8 metrics (N = 11,274) were also eliminated. The final analysis included 21,543 participants. **Figure 1** depicts the participant selection process.

### 2.2 Assessment of LE8

The LE8 scoring algorithm has 8 metrics, including 4 healthy behaviors (diet, PA, nicotine exposure, and sleep health) as well as 4 health factors (BMI, blood lipids and glucose, and BP). Each metric scores were 0–100 points, calculating the total LE8 score as the unweighted average of all 8 metric scores. As the AHA recommended, the LE8 score was assigned to three levels: low (0–49), moderate (50–79), and high CVH (80–100) (9).

The Healthy Eating Index (HEI)-2015 was deployed to evaluate diet metrics (19). The PA, smoking, and sleeping duration data were gathered by self-report questionnaires. In addition, the physical examination included measuring BP, height, and weight. The BMI was computed by dividing the weight (kg) by the height (m^2^). Blood samples were obtained and dispatched to laboratories for analyzing blood lipids, plasma glucose, and hemoglobin A1c levels. Additional comprehensive techniques for computing each metric LE8 score through NHANES data have been officially released (**Supplementary Table S1**) (9, 20).

### 2.3 Assessment of MetS

This study employed the National Cholesterol Education Program’s Adult Treatment Panel III report to define MetS (21). Individuals with MetS had three or more of these five criteria: 1) waist circumference ≥ 88 cm in women and ≥ 102 cm in men; 2) triglyceride ≥ 150 mg/dL or using drug treatment for elevated triglyceride; 3) high-density lipoprotein (HDL)-cholesterol level < 50 mg/dL for women and < 40 mg/dL for men or receiving drug for reduced HDL-cholesterol; 4) systolic blood pressure (SBP) ≥ 130 mmHg or diastolic blood pressure (DBP) ≥ 85 mmHg, or using drug for low HDL cholesterol; 5) fasting glucose ≥ 100 mg/dL or using antihypertensive medication.

### 2.4 Assessment of covariates

According to previous studies (22), several potential confounding variables were selected: age (20–39, 40–59, and 60–79); gender (male or female); race/ethnicity [non-Hispanic White, non-Hispanic Black, Mexican American, and other races (multi-racial and other Hispanic)]; education level (< 9th grade, 9th–11th grade (including 12th grade with no diploma), high school graduate (general educational development or equivalent), college graduate or above, and some college or associate’s degree); poverty income ratio (PIR; calculated as the ratio of monthly family income to poverty levels defined by Department of Health and Human Services guidelines: < 1.3, 1.3–3.5, and > 3.5); marital status (never married, widowed/divorced/separated, and married/living with partner). In addition, lifestyle and health variables were collected: alcohol consumption (never, former, and current) and energy intake (the total energy intake was expressed in quartiles), hypertension (an average SBP ≥ 140 mmHg and DBP ≥ 90 mmHg in 3 consecutive tests), CVD (a self-reported history of coronary heart disease, myocardial infarction, stroke, or angina by a trained health professional prior to the survey), depression, and DM (fasting plasma glucose ≥ 126 mg/dL, 2-h plasma glucose ≥ 200 mg/dL, hemoglobin A1c ≥ 6.5%, or self-reported DM by a professional doctor).

### 2.5 Statistical analyses

Due to the NHANES methodology, all the conducted statistical analyses were subject to weighting. One-way ANOVA analysis was deployed for comparing continuous variables by assessing their mean values together with their standard errors (SE). The chi-square test was utilized to examine counts and percentages of categorical data. The Pearson correlation coefficients were deployed for evaluating the pairwise correlation between 8 LE8 metrics. A survey-multivariable logistic regression model was employed to estimate the adjusted odds ratio (AOR) and 95% confidence interval (CI) for LE8 score associations with MetS (low CVH levels as the reference). Specifically, we used three different models: non-adjusted crude model, adjusted model 1 for age, gender, and race/ethnicity, and additionally adjusted model 2 for education level, marital status, PIR, and alcohol consumption. When the association of each LE8 metric score with MetS was evaluated, the remaining 7 LE8 components were further adjusted in model 2. In addition, our study deployed a restricted cubic spline model for examining the exposure-response relationship between LE8 scores and MetS. Furthermore, stratified analyses were conducted according to different demographic characteristics. To identify our findings’ robustness, we performed sensitivity analyses: 1) identifying LE8 score association with MetS in male and female populations; 2) adding covariates of survey cycle, DM, hypertension, CVD, depression, and total energy intake, respectively, to minimize their influences on the outcome; 3) setting high CVH (80–100) as the reference. Here, we conducted the statistical analyses in R language (X64 Version 4.3.1, R Foundation for Statistical Computing), with two-sided *P* < 0.05 representing statistical significance.

## 3 Results

### 3.1 Population characteristics

**Table 1** summarizes included participants’ characteristics by different CVH levels, as measured by LE8. The results showed that in seven survey cycles (2005–2018), a total of 21,543 sample represented 146.6 million non-institutionalized U.S. population at the age of 20–79 (**Figure 1**), with a weighted mean age of 46.38 (0.25) years and 10,961 (51.2%) being females. The total age-adjusted prevalence of MetS was 30.7% (0.52), with a higher prevalence among participants aged 60–79 years [47.4% (1.02)]. Furthermore, widowed/divorced/separated participants and those with a low PIR level (< 1.3) had a greater prevalence of MetS. The LE8 score of the participants was 68.58 (0.25) on average, and among them, 2,634 (9.7%), 14,508 (66.2%), and 4,401 (24.1%) had low, moderate, and high LE8-measured CVH, respectively. Participants with a higher LE8 score more likely to be younger, female, having a higher education level and income (PIR > 3.5), and married (all the *P* < 0.001), compared with those with a low CVH level. The results of pairwise correlation analysis showed a mild to moderate correlation among 8 LE8 metrics (**Supplementary Table S2**). There was no statistically significant correlation between nicotine exposure and blood glucose and BP. **Supplementary Tables S3–S5** present participants’ characteristics by gender, race/ethnicity, and MetS, respectively.

**Table 1:** Survey-weighted characteristics of the study population, NHANES 2005-2018 (n = 21,543).

Characteristics	Prevalence of age-adjusted MetS [(weighted % (SE))]	Total	LE8 score			*P-*value^a^
			Low CVH (0-49)	Moderate CVH (50-79)	High CVH (80-100)	
**Participants**	30.7 (0.52)	21,543 (100.0)	2,634 (9.7)	14,508 (66.2)	4,401 (24.1)	–
**Age (years)**	–	46.38 ± 0.25	52.52 ± 0.35	47.34 ± 0.26	41.25 ± 0.41	**< 0.001**
20-39	16.9 (0.66)	7,534 (36.3)	427 (18.8)	4,741 (33.8)	2,366 (50.5)	**< 0.001**
40-59	34.9 (0.76)	7,704 (40.5)	1,074 (46.5)	5,277 (41.5)	1,353 (35.2)	
60-79	47.4 (1.08)	6,305 (23.2)	1,133 (34.7)	4,490 (24.7)	682 (14.4)	
Gender						
Female	30.6 (0.68)	10,961 (51.2)	1,351 (52.3)	6,957 (47.9)	2,653 (59.9)	**< 0.001**
Male	30.8 (0.63)	10,582 (48.8)	1,283 (47.7)	7,551 (52.1)	1,748 (40.1)	
Race/ethnicity						
Non-Hispanic White	30.6 (0.65)	9,673 (70.8)	1,156 (67.8)	6,425 (70.2)	2,092 (73.6)	**< 0.001**
Non-Hispanic Black	28.0 (0.78)	4,471 (10.0)	772 (15.7)	3,154 (10.7)	545 (5.6)	
Mexican American	37.9 (0.97)	3,288 (7.7)	349 (7.0)	2,327 (8.0)	612 (7.0)	
Other races	29.3 (0.94)	4,111 (11.5)	357 (9.6)	2,602 (11.0)	1,152 (13.8)	
Education level						
Less than 9th grade	35.8 (1.45)	1,743 (3.9)	337 (7.8)	1,207 (4.0)	199 (2.2)	**< 0.001**
9-11th grade (including 12th grade with no diploma)	35.7 (1.07)	2,842 (9.5)	554 (17.3)	2,001 (10.3)	287 (4.1)	
High school graduate/GED or equivalent	36.3 (0.94)	4,896 (22.6)	741 (31.3)	3,565 (25.2)	590 (12.1)	
Some college or AA degree	32.7 (0.75)	6,621 (32.1)	742 (31.2)	4,600 (33.8)	1,279 (27.8)	
College graduate or above	22.3 (0.77)	5,441 (31.9)	260 (12.4)	3,135 (26.8)	2,046 (53.9)	
**PIR**	–	3.13 ± 0.04	3.55 ± 0.05	2.43 ± 0.06	3.08 ± 0.04	**< 0.001**
< 1.3	36.8 (0.81)	6,363 (19.1)	1,114 (31.6)	4,334 (19.4)	915 (13.0)	**< 0.001**
1.3-3.5	32.9 (0.66)	8,003 (34.8)	1,039 (40.5)	5,507 (36.1)	1,457 (29.0)	
> 3.5	26.7 (0.74)	7,177 (46.1)	481 (27.9)	4,667 (44.5)	2,029 (58.0)	
Marital status						
Never married	28.2 (1.46)	3,972 (17.5)	350 (13.0)	2,456 (15.9)	1,166 (24.0)	**< 0.001**
Widowed/Divorced/Separated	35.5 (1.11)	4,303 (16.6)	811 (26.1)	3,007 (17.8)	485 (9.6)	
Married/Living with partner	31.2 (0.59)	13,268 (65.8)	1,473 (60.9)	9,045 (66.3)	2,750 (66.4)	
Alcohol consumption						
Never	34.0 (1.13)	2,674 (9.6)	271 (8.0)	1,736 (9.2)	667 (11.4)	**< 0.001**
Former	39.9 (1.17)	3,396 (13.0)	723 (25.2)	2,322 (13.4)	351 (6.9)	
Current	28.6 (0.58)	15,473 (77.5)	1,640 (66.7)	10,450 (77.5)	3,383 (81.7)	
Hypertension						
Yes	51.5 (0.87)	8,650 (35.9)	1,918 (69.3)	6,188 (40.0)	544 (11.0)	**< 0.001**
No	18.5 (0.53)	12,893 (64.1)	716 (30.7)	8,320 (60.0)	3,857 (89.0)	
CVD						
Yes	53.1 (2.21)	1,960 (7.2)	595 (20.2)	1,256 (7.1)	109 (2.1)	**< 0.001**
No	29.0 (0.53)	19,583 (92.8)	2,039 (79.8)	13,252 (92.9)	4,292 (97.9)	
DM						
DM	74.1 (1.66)	3,360 (11.7)	1,126 (37.9)	2,158 (11.7)	76 (1.4)	**< 0.001**
IFG	61.8 (2.11)	986 (4.7)	154 (6.9)	728 (5.3)	104 (2.1)	
IGT	39.0 (2.03)	871 (3.6)	100 (3.9)	654 (4.1)	117 (2.2)	
No	22.0 (0.55)	16,326 (79.9)	1,254 (51.3)	10,968 (78.9)	4,104 (94.3)	
MetS						
Yes	–	7,215 (31.3)	1,862 (71.1)	5,099 (35.0)	254 (4.9)	**< 0.001**
No	–	14,328 (68.8)	772 (28.9)	9,409 (65.0)	4,147 (95.1)	
**Total energy intake (kcal)**	–	2,208.88 ± 9.56	2,135.89 ± 30.48	2,239.57 ± 11.50	2,154.15 ± 16.93	**< 0.001**
Q1 (< 1,462.0)	29.8 (0.88)	5,389 (21.9)	796 (26.1)	3,548 (21.5)	1,045 (21.5)	**< 0.001**
Q2 (1,462.0-1,971.0)	30.9 (0.83)	5,384 (24.7)	659 (24.8)	3,564 (24.1)	1,161 (26.3)	
Q3 (1,971.0-2,632.5)	31.1 (0.87)	5,384 (26.2)	602 (24.4)	3,622 (26.1)	1,160 (27.1)	
Q4 (> 2,632.5)	30.0 (0.87)	5,386 (27.2)	577 (24.7)	3,774 (28.4)	1,035 (24.0)	
Survey cycle						
2005-2006	27.9 (0.83)	2,876 (14.7)	352 (15.4)	2,116 (16.4)	408 (10.0)	**< 0.05**
2007-2008	32.9 (1.39)	3,353 (13.8)	439 (14.9)	2,281 (13.9)	633 (13.0)	
2009-2010	28.9 (0.96)	3,479 (13.9)	436 (13.8)	2,329 (13.6)	714 (14.6)	
2011-2012	28.9 (1.31)	3,064 (14.6)	376 (15.0)	1,980 (14.2)	708 (15.6)	
2013-2014	30.8 (1.40)	3,381 (15.3)	408 (16.0)	2,165 (14.5)	808 (17.3)	
2015-2016	34.3 (1.69)	3,053 (14.8)	370 (13.7)	2,053 (14.6)	630 (16.0)	
2017-2018	31.1 (1.70)	2,337 (12.8)	253 (11.2)	1,584 (12.8)	500 (13.5)	
LE8 metric scores						
Total	–	68.58 ± 0.25	41.55 ± 0.16	65.92 ± 0.11	86.83 ± 0.12	**< 0.001**
Diet	–	38.75 ± 0.50	18.90 ± 0.59	34.21 ± 0.46	59.26 ± 0.68	**< 0.001**
Physical activity	–	72.95 ± 0.50	27.26 ± 1.18	72.00 ± 0.55	94.04 ± 0.36	**< 0.001**
Nicotine exposure	–	70.98 ± 0.53	39.92 ± 1.18	67.81 ± 0.54	92.23 ± 0.44	**< 0.001**
Sleep health	–	83.64 ± 0.29	66.00 ± 0.83	82.94 ± 0.28	92.71 ± 0.31	**< 0.001**
Body mass index	–	60.15 ± 0.44	30.56 ± 0.79	55.39 ± 0.40	85.19 ± 0.45	**< 0.001**
Blood lipids	–	64.36 ± 0.35	41.82 ± 0.86	60.82 ± 0.40	83.20 ± 0.48	**< 0.001**
Blood glucose	–	86.82 ± 0.25	61.68 ± 0.70	86.49 ± 0.27	97.88 ± 0.19	**< 0.001**
Blood pressure	–	71.02 ± 0.34	46.25 ± 0.71	67.69 ± 0.37	90.18 ± 0.40	**< 0.001**

Footnotes: Continuous variables are presented as mean ± SE, and categorical variables are presented as n (weighted %).

^a^P-values were assessed by One-way ANOVA (continuous variables) or by Rao-Scott chi-square test (categorical variables). P-values shown in bold were statistically significant.

AA, Associate's Degree; CVD, Cardiovascular disease; CVH, Cardiovascular health; DM, Diabetes mellitus; GED, General educational development; IFG, Impaired fasting glycaemia; IGT, Impaired glucose tolerance; LE8, Life’s Essential 8; MetS, Metabolic syndrome; NHANES, National Health and Nutrition Examination Survey; PIR, Poverty income ratio; Q, Quartile; SE, Standard error.

### 3.2 Association between LE8 and MetS

**Figure 2:**
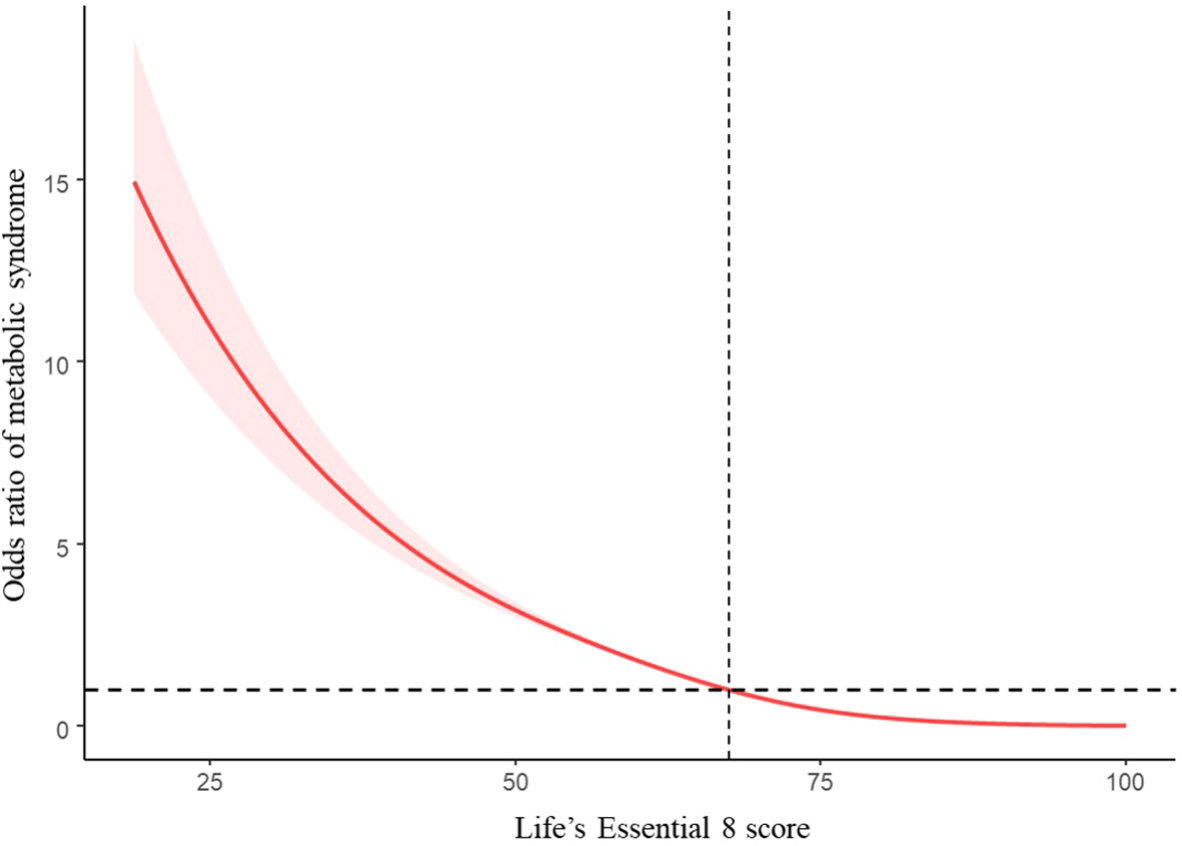
Restricted cubic spline and 95% CI between LE8 score and odd ratio of MetS, NHANES 2005–2018 (n = 21,354). The model adjusted for age, gender, race/ethnicity, education level, marital status, PIR, and alcohol consumption.

**Table 2** shows that participants who achieved a moderate (AOR = 0.234, 95% CI: 0.209, 0.262) or high LE8-evaluated CVH level (AOR = 0.026, 95% CI: 0.021, 0.032) had a lower risk of MetS after adjustment for potential covariates in comparison with those with a low CVH level. Furthermore, the total LE8 score and the odds ratio of MetS exhibited an inverse linear dose-response relationship (**Figure 2**; *P* for nonlinearity > 0.05). Similar trends (*P* for trend < 0.05) toward reduced risk of MetS were observed for participants with higher LE8 metric scores of diet (AOR = 0.858, 95% CI: 0.742, 0.993), nicotine exposure (AOR = 0.779, 95% CI: 0.681, 0.890), BMI (AOR = 0.054, 95% CI: 0.046, 0.064), blood lipids (AOR = 0.432, 95% CI: 0.386, 0.483), blood glucose (AOR = 0.089, 95% CI: 0.072, 0.109), and BP (AOR = 0.327, 95% CI: 0.280, 0.381). The LE8 metric scores of PA (AOR = 0.985, 95% CI: 0.871, 1.116) and sleep health (AOR = 0.981, 95% CI: 0.856, 1.125) did not have a significant inverse association with MetS.

**Table 2:** Association of LE8 scores with risk of MetS, NHANES 2005-2018 (n = 21,543).

	Cases/Participants	Crude model		Model 1		Model 2	
		COR (95% CI)	*P-*value	AOR (95% CI)	*P*-value	AOR (95% CI)	*P*-value
Total							
Low CVH (0-49)	1,862/2,634	Reference	–	Reference	–	Reference	–
Moderate CVH (50-79)	5,099/14,508	**0.219 (0.196, 0.244)**	**< 0.001**	**0.231 (0.207, 0.258)**	**< 0.001**	**0.234 (0.209, 0.262)**	**< 0.001**
High CVH (80-100)	254/4,401	**0.021 (0.017, 0.026)**	**< 0.001**	**0.024 (0.020, 0.030)**	**< 0.001**	**0.026 (0.021, 0.032)**	**< 0.001**
*P* for trend			**< 0.001**		**< 0.001**		**< 0.001**
Diet							
Low (0-49)	3,784/10,986	Reference	–	Reference	–	Reference	–
Moderate (50-79)	1,790/5,331	0.907 (0.819, 1.005)	0.062	**0.761 (0.685, 0.847)**	**< 0.001**	0.942 (0.816, 1.086)	0.406
High (80-100)	1,641/5,226	**0.743 (0.669, 0.826)**	**< 0.001**	**0.544 (0.487, 0.608)**	**< 0.001**	**0.858 (0.742, 0.993)**	**< 0.05**
*P* for trend			**< 0.001**		**< 0.001**		**< 0.05**
Physical activity							
Low (0-49)	2,628/6,428	Reference	–	Reference	–	Reference	–
Moderate (50-79)	356/1,033	**0.792 (0.668, 0.939)**	**< 0.05**	0.868 (0.723, 1.042)	0.126	1.021 (0.807, 1.292)	0.862
High (80-100)	4,231/14,082	**0.613 (0.560, 0.671)**	**< 0.001**	**0.704 (0.639, 0.777)**	**< 0.001**	0.985 (0.871, 1.116)	0.815
*P* for trend			**< 0.001**		**< 0.001**		0.794
Nicotine exposure							
Low (0-49)	1,624/5,036	Reference	–	Reference	–	Reference	–
Moderate (50-79)	1,939/4,603	**1.428 (1.274, 1.602)**	**< 0.001**	0.964 (0.848, 1.097)	0.577	**0.817 (0.697, 0.959)**	**< 0.05**
High (80-100)	3,630/11,847	**0.910 (0.832, 0.995)**	**< 0.05**	**0.831 (0.757, 0.913)**	**< 0.001**	**0.779 (0.681, 0.890)**	**< 0.001**
*P* for trend			**< 0.001**		**< 0.001**		**< 0.001**
Sleep health							
Low (0-49)	1,350/3,706	Reference	–	Reference	–	Reference	–
Moderate (50-79)	1,584/4,699	**0.818 (0.725, 0.924)**	**< 0.05**	**0.789 (0.690, 0.902)**	**< 0.001**	0.944 (0.787, 1.132)	0.527
High (80-100)	434/6,060	**0.808 (0.726, 0.899)**	**< 0.001**	**0.745 (0.664, 0.836)**	**< 0.001**	0.981 (0.856, 1.125)	0.785
*P* for trend			**< 0.001**		**< 0.001**		0.994
Body mass index							
Low (0-49)	4,779/8,438	Reference	–	Reference	–	Reference	–
Moderate (50-79)	2,002/7,045	**0.266 (0.239, 0.297)**	**< 0.001**	**0.234 (0.208, 0.263)**	**< 0.001**	**0.273 (0.241, 0.309)**	**< 0.001**
High (80-100)	434/6,060	**0.045 (0.038, 0.052)**	**< 0.001**	**0.042 (0.036, 0.049)**	**< 0.001**	**0.054 (0.046, 0.064)**	**< 0.001**
*P* for trend			**< 0.001**		**< 0.001**		**< 0.001**
Blood lipids							
Low (0-49)	3,505/7,276	Reference	–	Reference	–	Reference	–
Moderate (50-79)	1,375/5,089	**0.400 (0.359, 0.446)**	**< 0.001**	**0.464 (0.413, 0.520)**	**< 0.001**	**0.542 (0.476, 0.617)**	**< 0.001**
High (80-100)	2,335/9,178	**0.338 (0.306, 0.373)**	**< 0.001**	**0.390 (0.353, 0.430)**	**< 0.001**	**0.432 (0.386, 0.483)**	**< 0.001**
*P* for trend			**< 0.001**		**< 0.001**		**< 0.001**
Blood glucose							
Low (0-49)	2,184/2,719	Reference	–	Reference	–	Reference	–
Moderate (50-79)	2,086/4,341	**0.234 (0.198, 0.277)**	**< 0.001**	**0.243 (0.205, 0.287)**	**< 0.001**	**0.213 (0.174, 0.261)**	**< 0.001**
High (80-100)	2,945/14,483	**0.060 (0.050, 0.072)**	**< 0.001**	**0.073 (0.061, 0.088)**	**< 0.001**	**0.089 (0.072, 0.109)**	**< 0.001**
*P* for trend			**< 0.001**		**< 0.001**		**< 0.001**
Blood pressure							
Low (0-49)	1,982/3,459	Reference	–	Reference	–	Reference	–
Moderate (50-79)	2,332/7,116	**0.325 (0.284, 0.371)**	**< 0.001**	**0.396 (0.343, 0.458)**	**< 0.001**	**0.399 (0.342, 0.465)**	**< 0.001**
High (80-100)	2,190/9,904	**0.182 (0.161, 0.205)**	**< 0.001**	**0.249 (0.219, 0.283)**	**< 0.001**	**0.327 (0.280, 0.381)**	**< 0.001**
*P* for trend			**< 0.001**		**< 0.001**		**< 0.001**

For the total LE8 score: The crude model was unadjusted. Model 1 was adjusted for age, gender, and race/ethnicity. Model 2 was adjusted for age, gender, race/ethnicity, education level, marital status, PIR, and alcohol consumption. For the 8 LE8 metrics scores: Model 2 adjusted for gender, age, race/ethnicity, education level, marital status, PIR, alcohol consumption, diet, nicotine exposure, physical activity, sleep health, body mass index, blood glucose, blood lipids, and blood pressure. When the association between each LE8 metric and the incidence of MetS was evaluated, this metric was excluded from the adjustment. The results of COR (95% CI), AOR (95% CI), and *P*-value shown in bold were statistically significant. *P*-value < 0.05 or *P*-value < 0.001.

AOR, Adjusted odds ratio; CI, Confidence interval; COR, Crude odds ratio; CVH, Cardiovascular health; LE8, Life’s Essential 8; MetS, Metabolic syndrome; NHANES, National Health and Nutrition Examination Survey; PIR, Poverty income ratio.

### 3.3 Stratified and sensitivity analysis

**Table 3** represents the stratified analysis results. In our stratified analysis by gender, age, race/ethnicity, education level, PIR, marital status, and alcohol consumption, participants who achieved a moderate or high LE8-evaluated CVH levels in all subgroups showed the decreased risk of MetS relative to those with a low CVH level. Moreover, we observed a significant interaction between gender, marital status, and LE8 (*P* for interaction < 0.05).

**Table 3:** Association of LE8 score with risk of MetS in stratified analyses, NHANES 2005-2018 (n = 21,543).

Subgroup	LE8 score					*P-*interaction
	Low CVH (0-49)	Moderate CVH (50-79)		High CVH (80-100)		
		AOR (95% CI)	*P*-value	AOR (95% CI)	*P*-value	
Gender						
Female	Reference	**0.231 (0.194, 0.274)**	**< 0.001**	**0.024 (0.019, 0.031)**	**< 0.001**	**< 0.05**
Male	Reference	**0.264 (0.216, 0.323)**	**< 0.001**	**0.034 (0.023, 0.050)**	**< 0.001**	
Age (years)						
20-39	Reference	**0.242 (0.183, 0.320)**	**< 0.001**	**0.022 (0.015, 0.032)**	**< 0.001**	0.386
40-59	Reference	**0.246 (0.206, 0.295)**	**< 0.001**	**0.028 (0.020, 0.040)**	**< 0.001**	
60-79	Reference	**0.276 (0.216, 0.353)**	**< 0.001**	**0.040 (0.028, 0.057)**	**< 0.001**	
Race/ethnicity						
Non-Hispanic White	Reference	**0.251 (0.212, 0.298)**	**< 0.001**	**0.027 (0.020, 0.036)**	**< 0.001**	0.055
Non-Hispanic Black	Reference	**0.191 (0.156, 0.231)**	**< 0.001**	**0.023 (0.013, 0.040)**	**< 0.001**	
Mexican American	Reference	**0.273 (0.198, 0.377)**	**< 0.001**	**0.046 (0.030, 0.070)**	**< 0.001**	
Other races	Reference	**0.288 (0.209, 0.396)**	**< 0.001**	**0.037 (0.024, 0.056)**	**< 0.001**	
Education level						
Less than 9th grade	Reference	**0.276 (0.192, 0.397)**	**< 0.001**	**0.021 (0.010, 0.046)**	**< 0.001**	0.132
9-11th grade (including 12th grade with no diploma)	Reference	**0.280 (0.214, 0.366)**	**< 0.001**	**0.030 (0.015, 0.062)**	**< 0.001**	
High school graduate/GED or equivalent	Reference	**0.229 (0.181, 0.291)**	**< 0.001**	**0.043 (0.025, 0.072)**	**< 0.001**	
Some college or AA degree	Reference	**0.257 (0.202, 0.327)**	**< 0.001**	**0.028 (0.019, 0.041)**	**< 0.001**	
College graduate or above	Reference	**0.202 (0.134, 0.303)**	**< 0.001**	**0.021 (0.014, 0.032)**	**< 0.001**	
Marital status						
Never married	Reference	**0.179 (0.127, 0.252)**	**< 0.001**	**0.018 (0.010, 0.033)**	**< 0.001**	**< 0.05**
Widowed/Divorced/Separated	Reference	**0.265 (0.203, 0.346)**	**< 0.001**	**0.012 (0.007, 0.023)**	**< 0.001**	
Married/Living with partner	Reference	**0.262 (0.225, 0.306)**	**< 0.001**	**0.034 (0.026, 0.045)**	**< 0.001**	
PIR						
< 1.3	Reference	**0.213 (0.169, 0.269)**	**< 0.001**	**0.030 (0.020, 0.046)**	**< 0.001**	0.462
1.3-3.5	Reference	**0.259 (0.214, 0.312)**	**< 0.001**	**0.026 (0.019, 0.037)**	**< 0.001**	
> 3.5	Reference	**0.267 (0.194, 0.366)**	**< 0.001**	**0.031 (0.020, 0.048)**	**< 0.001**	
Alcohol consumption						
Never	Reference	**0.237 (0.203, 0.277)**	**< 0.001**	**0.026 (0.020, 0.035)**	**< 0.001**	0.564
Former	Reference	**0.294 (0.234, 0.369)**	**< 0.001**	**0.033 (0.018, 0.059)**	**< 0.001**	
Current	Reference	**0.212 (0.137, 0.327)**	**< 0.001**	**0.025 (0.014, 0.045)**	**< 0.001**	

The multivariable logistic regression model was adjusted for age, gender, race/ethnicity, education level, marital status, PIR, and alcohol consumption. The results of AOR (95% CI), *P*-interaction, and *P*-value shown in bold were statistically significant. *P*-value < 0.05 or *P*-value < 0.001.

AA, Associate’s Degree; AOR, Adjusted odds ratio; CI, Confidence interval; CVH, Cardiovascular health; GED, General educational development; LE8, Life’s Essential 8; MetS, Metabolic syndrome; NHANES, National Health and Nutrition Examination Survey; PIR, Poverty income ratio.

The results of the sensitivity analysis aligned with our results. **Supplementary Tables S6, S7** demonstrate that the association between LE8-evaluated CVH and decreased risks of MetS in both male and female populations remained robust. Moreover, even after additional adjustments for the survey cycle, DM, hypertension, CVD, depression, and total energy intake, the high CVH level was still significantly related to a lower risk of MetS (**Supplementary Table S8**). Finally, when the high CVH group was used as the reference, participants with a low CVH had a higher risk of MetS (**Supplementary Table S9**).

## 4 Discussion

This nationally representative study of the U.S. population showed that LE8 and its metric scores had an inverse association with MetS. Our results remained robust after stratified and sensitivity analyses. These findings suggested that the potential beneficial impacts of maintaining a higher CVH level on preventing and managing MetS. Given the modifiable nature of several LE8 components, the LE8 guidelines may serve as a plausible prevention approach for MetS, which provides significant insights for caregivers and clinical staff. Moreover, LE8 as a comprehensive indicator, may be helpful for the risk assessment of MetS and the screening of potential high‐risk populations.

Our study found that individuals with a higher LE8 score exhibited a substantially diminished risk of MetS in comparison to those with a low CVH level, consistent with relevant prior studies. A study has revealed a statistically significant disparity in the presence of MetS among individuals with LS7-measured poor, intermediate, and ideal CVH in terms of MetS presence (*P* < 0.001). Moreover, the poor [hazard ratio (HR): 1.83, 95 % CI: 1.08–3.10] and intermediate CVH (HR: 1.57, 95% CI: 1.34–1.84) individuals with MetS exhibited a higher CVD risk (23). Another prospective study of 341,331 participants from the UK Biobank has demonstrated that the ideal CVH group, in comparison to the poor CVH group, mitigated the mortality risk associated with cardiometabolic diseases by approximately 62% for males and 53% for females (24). Despite there are some limitations of LS7 (10), the findings indicated that individuals with ideal CVH status may have lower risks of MetS and CVD (23, 24). One study compromising 170,726 participants from the UK Biobank has estimated the LE8-evaluated CVH association and the risk of 44 common non-communicable chronic diseases (25). In comparison with the low CVH group, the high CVH group had an 84% decreased risk of non-communicable chronic diseases in metabolic systems (HR: 0.16, 95% CI: 0.15–0.18) (25). Several potential mechanisms may explain the inverse association between LE8 and MetS. First, sustaining a better CVH and preventing MetS share common influencing factors related to the health behaviors of LE8, as well as health factors that are determinants of MetS (26–31). For instance, engaging in adequate and regular PA could improve the levels of cytokines related to MetS, including CRP, TNF-α, and IL-8/10 (32), and mitigate systemic inflammation by promoting anti-inflammatory adipokine release to reduce MetS risk (33, 34). Furthermore, the protective effects of LE8 in mitigating the risk of MetS could be explained by the physiological mechanism that appropriate weight reduction lowered free fatty acids and improved insulin resistance status, preventing MetS development (35). After controlling confounding and potential variables, including DM, hypertension, CVD, depression, and total energy intake, the results remained stable, indicating a higher LE8 score had potential protective impacts on MetS. Accordingly, our study exhibited lower heterogeneity besides representing more reliable main findings that individuals with a higher LE8 score were at a lower MetS risk.

Our study also found that, in addition to health factors, single nicotine exposure or diet metric scores of LE8 were significantly associated with the risk of MetS, in line with previous studies (36, 37). The pathophysiological mechanism shows that smoking can potentially stimulate lipolysis, releasing free fatty acids that may detrimentally impact fasting blood sugar levels through the impairment of pancreatic cells (37). In addition, an unhealthy diet may cause mitochondrial dysfunction, which can result in oxidative stress, bioenergy depletion, protein accumulation, and cell death. All these factors are related to MetS pathogenesis (38). Nonetheless, not all the LE8 metrics were involved in the risk of MetS. This study did not observe significantly inverse associations between PA, sleep health, and MetS, which inconsistent with previous studies (27, 28). Several reasons might explain the discrepancy. First, the PA and sleep duration measurements were obtained through self-report rather than objective measurement, which may have caused measurement errors that affected the reliability of relationships between PA, sleep metrics and MetS. Second, in addition to sleep duration, sleep quality also plays an important role in MetS that was not covered by LE8 (39). Further works are required to understand better the mechanism of the PA, sleep and MetS risks.

To our knowledge, this is the first research to examine the association between LE8 and MetS in representative general adults. Although, Yang et al. examined the association of MUH with LE8 (13). However, in that study, adults with one of the four MetS components were classified as MUH, which was different from the definition of MetS and could not fully reflect metabolic health. Additionally, we further explored the dose-response relationship in associations between LE8 scores and MetS. However, this study has some constraints. First, due to the of cross-sectional study design, we were unable to conclude a causal relationship between LE8 and MetS. However, the health factors of LE8 partly overlap the diagnostic criteria of MetS, implying that reverse causality is less likely to occur in our study. Therefore, high-quality prospective studies should be conducted to verify this causal relationship in the future. Second, health behavior metrics were measured by self-report questionnaires, which are subject to recall and social desirability biases and may have some impacts on the presented study results. Third, four metrics in the LE8 are components of MetS, which may affect the validity of the relationship between a low LE8-evaluated CVH level and MetS. It is, therefore, necessary to interpret with caution the association of a low LE8-evaluated CVH level with MetS. Finally, although we adjusted several potential confounders, such as energy intake, CVD, and so on, and conducted sensitivity analyses, it is undeniable that there are some unknown potential confounding factors (genetic factors, etc.) that have not been accounted.

## 5 Conclusions

In summary, LE8 was inversely associated with the risk of MetS among a national, large sample of U.S. adults. Adhering to LE8 guidelines to sustain a higher CVH level may be beneficial for preventing MetS. Future studies are required to further examine this causal relationship.

## Data Availability

Publicly available datasets were analyzed in this study. This data can be found here: https://doi.org/10.6084/m9.figshare.26927497.v4.
