# Supplementary material for: The inverse relationship between Life’s Essential 8 and risk of metabolic syndrome: evidence from NHANES 2005-2018
Source: Front Endocrinol (Lausanne). 2024 Oct 28;15:1449930. doi: 10.3389/fendo.2024.1449930 (PMC11551013; doi:10.3389/fendo.2024.1449930)
Supplement: Supplementary file 1 [file Table1.docx]

Supplementary Materials

The inverse relationship between Life’s essential 8 and risk of metabolic syndrome: evidence from NHANES 2005-2018

**Table of Contents**

[**Table S1** **Pairwise correlation of the LE8 metrics, NHANES 2005-2018 (n = 21,543).** 3](#_Toc169445559)

[**Table S2** **Definition and scoring approach for the American Heart Association’s Life’s Essential 8.** 4](#_Toc169445560)

[**Table S3** **Survey-weighted characteristic of the study population by gender, NHANES 2005-2018 (n = 21,543).** 6](#_Toc169445561)

[**Table S4 Survey-weighted characteristic of the study population by race/ethnicity, NHANES 2005-2018 (n = 21,543).** 8](#_Toc169445562)

[**Table S5 Survey-weighted characteristic of the study population by MetS status, NHANES 2005-2018 (n = 21,543).** 11](#_Toc169445563)

[**Table S6 Association of LE8 scores with the risk of MetS in the female population, NHANES 2005-2018 (n = 10,961).** 14](#_Toc169445564)

[**Table S7 Association of LE8 scores with the risk of MetS in the male population, NHANES 2005-2018 (n = 10,582).** 16](#_Toc169445565)

[**Table S8 Association of LE8 score with the risk of MetS for additional adjustments, NHANES 2005-2018 (n = 21,543).** 18](#_Toc169445566)

[**Table S9 Association of LE8 score with the risk of MetS, High CVH (80-100) as the reference, NHANES 2005–2018 (n = 21,543).** 19](#_Toc169445567)

| **Table S1** **Pairwise correlation of the LE8 metrics, NHANES 2005-2018 (n = 21,543).** | | | | | | | | |
| --- | --- | --- | --- | --- | --- | --- | --- | --- |
| **LE8 metrics** | **Diet** | **PA** | **Nicotine exposure** | **Sleep health** | **BMI** | **Blood lipids** | **Blood glucose** | **BP** |
| **Diet** | 1.00 |  |  |  |  |  |  |  |
| **PA** | **0.07^*^** | 1.00 |  |  |  |  |  |  |
| **Nicotine exposure** | **0.22^**^** | 0.01 | 1.00 |  |  |  |  |  |
| **Sleep health** | **0.10^**^** | **0.04^**^** | **0.11^**^** | 1.00 |  |  |  |  |
| **BMI** | **0.10^**^** | **0.10^**^** | **-0.06^**^** | **0.06^**^** | 1.00 |  |  |  |
| **Blood lipids** | **0.02^**^** | **0.05^**^** | **0.04^**^** | **0.02^*^** | **0.17^**^** | 1.00 |  |  |
| **Blood glucose** | **-0.02^*^** | **0.14^**^** | 0.00 | **0.06^**^** | **0.26^**^** | **0.14^**^** | 1.00 |  |
| **BP** | **-0.03^**^** | **0.09^**^** | 0.00 | **0.04^**^** | **0.21^**^** | **0.18^**^** | **0.30^**^** | 1.00 |
| Footnotes: Correlation coefficients were in bold indicating statistically significant correlation.  * *P* < 0.05, ** *P* < 0.001.  Abbreviations: BP, Blood pressure; BMI, Body mass index; LE8, Life’s essential 8; PA, Physical activity; NHANES: National Health and Nutrition Examination Survey. | | | | | | | | |

| **Table S2** **Definition and scoring approach for the American Heart Association’s Life’s Essential 8.** | | | |
| --- | --- | --- | --- |
| **Domain** | **CVH metric** | **Method of measurement** | **Quantification and scoring of CVH metric: adults (≥ 20 years of age)** |
| **Health behaviors** | Diet | Healthy Eating Index-2015 diet score percentile | Quantiles of HEI-2015 (population)  Scoring (Population):  Points Quantile  100 ≥ 95th percentile (top/ideal diet)  80 75th-94th percentile  50 50th-74th percentile  25 25th-49th percentile  0 1st-24th percentile (bottom/least ideal quartile) |
|  | Physical activity | Self-reported minutes of moderate or vigorous physical activity per week | Metric: Minutes of moderate (or greater) intensity activity per week  Scoring:  Points Minutes  100 ≥ 150  90 120-149  80 90-119  60 60-89  40 30-59  20 1-29  0 0 |
|  | Nicotine exposure | Self-reported use of cigarettes or inhaled NDS | Metric: Combustible tobacco use and/or inhaled NDS use; or secondhand smoke exposure  Scoring:  Points Status  100 Never smoker  75 Former smoker, quit ≥5 years  50 Former smoker, quit 1-< 5 years  25 Former smoker, quit < 1 year, or currently using inhaled NDS  0 Current smoker  Subtract 20 points (unless score is 0) for living with active indoor smoker in home |
|  | Sleep health | Self-reported average hours of sleep per night | Metric: Average hours of sleep per night  Scoring:  Points Level  100 7-< 9  90 9-< 10  70 6-< 7  40 5-< 6 or ≥ 10  20 4-< 5  0 < 4 |
| **Health factors** | Body mass index | Body weight (kg) divided by height squared (m^2^) | Metric: Body mass index (kg/m^2^)  Scoring:  Points Level  100 < 25  70 25.0-29.9  30 30.0-34.9  15 35.0-39.9  0 ≥ 40.0 |
|  | Blood lipids | Plasma total and HDL-cholesterol with calculation of non-HDL-cholesterol | Metric: Non-HDL-cholesterol (mg/dL)  Scoring:  Points Level  100 < 130  60 130-159  40 160-189  20 190-219  0 ≥ 220  If drug-treated level, subtract 20 points |
|  | Blood glucose | Fasting blood glucose or casual hemoglobin A1c | Metric: Fasting blood glucose (mg/dL) or hemoglobin A1c (%)  Scoring:  Points Level  100 No history of diabetes and FBG < 100 (or HbA1c < 5.7)  60 No diabetes and FBG 100 – 125 (or HbA1c 5.7-6.4) (Pre-diabetes)  40 Diabetes with HbA1c < 7.0  30 Diabetes with HbA1c 7.0-7.9  20 Diabetes with HbA1c 8.0-8.9  10 Diabetes with Hb A1c 9.0-9.9  0 Diabetes with HbA1c ≥ 10.0 |
|  | Blood pressure | Appropriately measured systolic and diastolic blood pressure | Metric: Systolic and diastolic blood pressure (mm Hg)  Scoring:  Points Level  100 < 120/< 80 (Optimal)  75 120-129/< 80 (Elevated)  50 130-139 or 80-89 (Stage 1 hypertension)  25 140-159 or 90-99  0 ≥ 160 or ≥ 100  Subtract 20 points if treated level |
| References:  [1] Lloyd-Jones DM, Hong Y, Labarthe D, et al. Defining and setting national goals for cardiovascular health promotion and disease reduction: the American Heart Association's strategic Impact Goal through 2020 and beyond. Circulation. 2010;121(4):586-613. doi:10.1161/CIRCULATIONAHA.109.192703  [2] Lloyd-Jones DM, Allen NB, Anderson CAM, et al. Life's Essential 8: Updating and Enhancing the American Heart Association's Construct of Cardiovascular Health: A Presidential Advisory From the American Heart Association. Circulation. 2022;146(5):e18-e43. doi:10.1161/CIR.0000000000001078  Abbreviations: CVH, Cardiovascular health; FBG, Fasting blood glucose; HbA1c: Hemoglobin A1c; HDL, High-density lipoprotein; NDS, Nicotine-delivery system; HEI, Healthy Eating Index. | | | |

| **Table S3** **Survey-weighted characteristic of the study population by gender, NHANES 2005-2018 (n = 21,543).** | | | | |
| --- | --- | --- | --- | --- |
| **Characteristics** | **Total** | **Gender** | | ***P*-value^a^** |
|  |  | **Female** | **Male** |  |
| **Age (years)** | 46.38 ± 0.25 | 46.99 ± 0.27 | 45.74 ± 0.27 | **< 0.001** |
| 20-39 | 7,534 (36.3) | 3,758 (34.6) | 3,776 (38.2) | **< 0.001** |
| 40-59 | 7,704 (40.5) | 4,046 (40.9) | 3,658 (40.0) |  |
| 60-79 | 6,305 (23.2) | 3,157 (24.5) | 3,148 (21.8) |  |
| **Race/ethnicity** |  |  |  |  |
| Non-Hispanic White | 9,673 (70.8) | 4,843 (70.6) | 4,830 (71.1) | **< 0.001** |
| Non-Hispanic Black | 4,471 (10.0) | 2,320 (10.8) | 2,151 (9.1) |  |
| Mexican American | 3,288 (7.7) | 1,673 (7.1) | 1,615 (8.3) |  |
| Other races | 4,111 (11.5) | 2,125 (11.6) | 1,986 (11.5) |  |
| **Education level** |  |  |  |  |
| Less than 9th grade | 1,743 (3.9) | 816 (3.5) | 927 (4.4) | **< 0.001** |
| 9-11th grade (including 12th grade with no diploma) | 2,842 (9.5) | 1,382 (9.0) | 1,460 (9.9) |  |
| High school graduate /GED or equivalent | 4,896 (22.6) | 2,369 (21.7) | 2,527 (23.7) |  |
| Some college or AA degree | 6,621 (32.1) | 3,637 (34.0) | 2,984 (30.0) |  |
| College graduate or above | 5,441 (31.9) | 2,757 (31.8) | 2,684 (32.1) |  |
| **PIR** | 3.13 ± 0.04 | 3.05 ± 0.04 | 3.21 ± 0.04 | **< 0.001** |
| < 1.3 | 6,363 (19.1) | 3,416 (20.4) | 2,947 (17.6) | **< 0.001** |
| 1.3-3 | 8,003 (34.8) | 4,070 (35.3) | 3,933 (34.3) |  |
| > 3.5 | 7,177 (46.1) | 3,475 (44.3) | 3,702 (48.1) |  |
| **Marital status** |  |  |  |  |
| Never married | 3,972 (17.5) | 1,974 (16.0) | 1,998 (19.2) | **< 0.001** |
| Widowed/Divorced/Separated | 4,303 (16.7) | 2,800 (21.5) | 1,503 (11.6) |  |
| Married/Living with partner | 13,268 (65.8) | 6,187 (62.5) | 7,081 (69.3) |  |
| **Alcohol consumption** |  |  |  |  |
| Current | 15,473 (77.5) | 7,358 (74.3) | 8,115 (80.8) | **< 0.001** |
| Former | 3,396 (13.0) | 1,667 (12.8) | 1,729 (13.1) |  |
| Never | 2,674 (9.6) | 1,936 (12.9) | 738 (6.1) |  |
| **Hypertension** |  |  |  |  |
| Yes | 8,650 (35.9) | 4,276 (34.5) | 4,374 (37.3) | **< 0.001** |
| No | 12,893 (64.1) | 6,685 (65.5) | 6,208 (62.7) |  |
| **CVD** |  |  |  |  |
| Yes | 1,960 (7.2) | 829 (6.1) | 1,131 (8.3) | **< 0.001** |
| No | 19,583 (92.8) | 10,132 (93.9) | 9,451 (91.7) |  |
| **DM** |  |  |  |  |
| DM | 3,360 (11.7) | 1,626 (11.1) | 1,734 (12.4) | **< 0.001** |
| IFG | 986 (4.7) | 371 (3.6) | 615 (5.8) |  |
| IGT | 871 (3.7) | 480 (3.9) | 391 (3.4) |  |
| No | 16,326 (79.9) | 8,484 (81.5) | 7,842 (78.4) |  |
| **MetS** |  |  |  |  |
| Yes | 7,215 (31.3) | 3,855 (31.6) | 3,360 (30.9) | 0.409 |
| No | 14,328 (68.7) | 7,106 (68.4) | 7,222 (69.1) |  |
| **Total energy intake (kcal)** | 2,208.88 ± 9.56 | 1,850.46 ± 9.18 | 2,584.76 ± 12.76 | **< 0.001** |
| Q1 (< 1,462.0) | 5,389 (21.9) | 3,839 (31.8) | 1,550 (11.6) | **< 0.001** |
| Q2 (1,462.0-1,971.0) | 5,384 (24.7) | 3,234 (30.6) | 2,150 (18.5) |  |
| Q3 (1,971.0-2,632.5) | 5,384 (26.2) | 2,505 (24.3) | 2,879 (28.2) |  |
| Q4 (> 2,632.5) | 5,386 (27.2) | 1,383 (13.3) | 4,003 (41.8) |  |
| **Survey cycle** |  |  |  |  |
| 2005-2006 | 2,876 (14.7) | 1,415 (14.7) | 1,461 (14.8) | 0.860 |
| 2007-2008 | 3,353 (13.8) | 1,726 (14.1) | 1,627 (13.5) |  |
| 2009-2010 | 3,479 (13.9) | 1,762 (13.7) | 1,717 (14.0) |  |
| 2011-2012 | 3,064 (14.6) | 1,522 (14.5) | 1,542 (14.8) |  |
| 2013-2014 | 3,381 (15.3) | 1,767 (15.3) | 1,614 (15.3) |  |
| 2015-2016 | 3,053 (14.8) | 1,559 (14.8) | 1,494 (14.8) |  |
| 2017-2018 | 2,337 (12.8) | 1,210 (12.9) | 1,127 (12.7) |  |
| **LE8 metric scores** | | | |  |
| Total | 68.58 ± 0.25 | 69.62 ± 0.30 | 67.50 ± 0.26 | **< 0.001** |
| Diet | 38.75 ± 0.50 | 41.94 ± 0.60 | 35.41 ± 0.53 | **< 0.001** |
| Physical activity | 72.95 ± 0.50 | 68.70 ± 0.69 | 77.41 ± 0.53 | **< 0.001** |
| Nicotine exposure | 70.98 ± 0.53 | 74.24 ± 0.59 | 67.56 ± 0.62 | **< 0.001** |
| Sleep health | 83.64 ± 0.29 | 84.00 ± 0.36 | 83.27 ± 0.34 | 0.071 |
| Body mass index | 60.15 ± 0.44 | 60.26 ± 0.56 | 60.03 ± 0.50 | 0.705 |
| Blood lipids | 64.36 ± 0.35 | 66.58 ± 0.45 | 62.03 ± 0.44 | **< 0.001** |
| Blood glucose | 86.82 ± 0.25 | 87.46 ± 0.31 | 86.14 ± 0.32 | **< 0.001** |
| Blood pressure | 71.02 ± 0.34 | 73.79 ± 0.36 | 68.12 ± 0.47 | **< 0.001** |
| Footnotes: Continuous variables are presented as mean ± SE, and categorical variables are presented as n (weighted %).  ^a^ The *P*-values were assessed by *T*-test (continuous variables) or by Rao-Scott chi-square test (categorical variables). *P*-values presented with bold values were statistically significant.  Abbreviations: AA, Associate's Degree; CVD, Cardiovascular disease; DM, Diabetes mellitus; GED, General educational development; IFG, Impaired fasting glycaemia; IGT, Impaired glucose tolerance; LE8, Life’s Essential 8; MetS, Metabolic syndrome; NHANES, National Health and Nutrition Examination Survey; PIR, Poverty income ratio; Q, Quartile; SE, Standard error. | | | | |

| **Table S4 Survey-weighted characteristic of the study population by race/ethnicity, NHANES 2005-2018 (n = 21,543).** | | | | | | |
| --- | --- | --- | --- | --- | --- | --- |
| **Characteristics** | **Total** | **Race/ethnicity** | | | | ***P*-value^a^** |
|  |  | **Non-Hispanic White** | **Non-Hispanic Black** | **Mexican American** | **Other races** |  |
| **Age (years)** | 46.38 ± 0.25 | 40.05 ± 0.37 | 43.95 ± 0.36 | 48.07 ± 0.29 | 42.31 ± 0.36 | **< 0.001** |
| 20-39 | 7,534 (36.3) | 1,309 (53.8) | 1,494 (41.7) | 3,099 (31.8) | 1,632 (48.0) | **< 0.001** |
| 40-59 | 7,704 (40.5) | 1,152 (35.0) | 1,623 (40.4) | 3,461 (41.6) | 1,468 (37.3) |  |
| 60-79 | 6,305 (23.2) | 827 (11.2) | 1,354 (17.9) | 3,113 (26.6) | 1,011 (14.7) |  |
| **Gender** |  |  |  |  |  |  |
| Female | 10,961 (51.2) | 1,673 (47.3) | 2,320 (55.3) | 4,843 (51.0) | 2,125 (51.3) | **< 0.001** |
| Male | 10,582 (48.8) | 1,615 (52.7) | 2,151 (44.7) | 4,830 (49.0) | 1,986 (48.7) |  |
| **Education level** |  |  |  |  |  |  |
| Less than 9th grade | 1,743 (3.9) | 917 (21.7) | 157 (2.7) | 284 (1.7) | 385 (6.9) | **< 0.001** |
| 9-11th grade (including 12th  grade with no diploma) | 2,842 (9.5) | 653 (20.0) | 742 (15.4) | 1,003 (7.4) | 444 (9.7) |  |
| High school graduate/GED or  equivalent | 4,896 (22.6) | 682 (23.1) | 1,159 (26.3) | 2,372 (22.8) | 683 (18.0) |  |
| Some college or AA degree | 6,621 (32.1) | 727 (24.5) | 1,563 (35.7) | 3,176 (32.6) | 1,155 (30.7) |  |
| College graduate or above | 5,441 (31.9) | 309 (10.7) | 850 (20.0) | 2,838 (35.4) | 1,444 (34.7) |  |
| **PIR** | 3.13 ± 0.04 | 2.03 ± 0.05 | 2.39 ± 0.05 | 3.41 ± 0.04 | 2.76 ± 0.06 | **< 0.001** |
| < 1.3 | 6,363 (19.1) | 1,395 (41.1) | 1,408 (31.7) | 2,358 (13.8) | 1,202 (25.7) | **< 0.001** |
| 1.3-3.5 | 8,003 (34.8) | 1,324 (40.3) | 1,861 (41.7) | 3,326 (32.8) | 1,492 (37.8) |  |
| > 3.5 | 7,177 (46.1) | 569 (18.6) | 1,202 (26.6) | 3,989 (53.4) | 1,417 (36.5) |  |
| **Marital status** |  |  |  |  |  |  |
| Never married | 3,972 (17.5) | 462 (17.7) | 1,291 (33.0) | 1,400 (14.6) | 819 (21.9) | **< 0.001** |
| Widowed/Divorced/Separated | 4,303 (16.6) | 554 (13.3) | 1,147 (22.6) | 1,911 (16.5) | 691 (14.5) |  |
| Married/Living with partner | 13,268 (65.8) | 2,272 (69.0) | 2,033 (44.4) | 6,362 (68.8) | 2,601 (63.6) |  |
| **Alcohol consumption** |  |  |  |  |  |  |
| Current | 15,473 (77.5) | 2,236 (73.7) | 3,052 (71.3) | 7,314 (79.6) | 2,871 (72.3) | **< 0.001** |
| Former | 3,396 (13.0) | 535 (12.9) | 782 (14.1) | 1,553 (12.9) | 526 (12.4) |  |
| Never | 2,674 (9.6) | 517 (13.4) | 637 (14.6) | 806 (7.5) | 714 (15.4) |  |
| **Hypertension** |  |  |  |  |  |  |
| Yes | 8,650 (35.9) | 1,098 (25.8) | 2,281 (44.8) | 3,856 (36.6) | 1,415 (30.4) | **< 0.001** |
| No | 12,893 (64.1) | 2,190 (74.2) | 2,190 (55.2) | 5,817 (63.4) | 2,696 (69.6) |  |
| **CVD** |  |  |  |  |  |  |
| Yes | 1,960 (7.2) | 188 (3.9) | 480 (8.5) | 1,018 (7.6) | 274 (5.7) | **< 0.001** |
| No | 19,583 (92.8) | 3,100 (96.1) | 3,991 (91.5) | 8,655 (92.4) | 3,837 (94.3) |  |
| **DM** |  |  |  |  |  |  |
| DM | 3,360 (11.7) | 609 (13.0) | 873 (15.9) | 1,269 (11.0) | 609 (11.9) | **< 0.001** |
| IFG | 986 (4.7) | 172 (5.2) | 150 (3.2) | 485 (4.9) | 179 (4.2) |  |
| IGT | 871 (3.6) | 166 (4.3) | 132 (2.7) | 398 (3.7) | 175 (3.9) |  |
| No | 16,326 (79.9) | 2,341 (77.5) | 3,316 (78.2) | 7,521 (80.4) | 3,148 (80.0) |  |
| **MetS** |  |  |  |  |  |  |
| Yes | 7,215 (31.3) | 1,246 (33.5) | 1,338 (26.9) | 3,431 (32.4) | 1,200 (26.4) | **< 0.001** |
| No | 14,328 (68.7) | 2,042 (66.5) | 3,133 (73.1) | 6,242 (67.6) | 2,911 (73.6) |  |
| **Total energy intake (kcal)** | 2,208.88 ± 9.56 | 2,276.87 ± 23.11 | 2,146.08 ± 17.63 | 2,225.31 ± 11.58 | 2,117.15 ± 21.51 | **< 0.001** |
| Q1 (< 1,462.0) | 5,389 (21.9) | 809 (21.6) | 1,243 (26.3) | 2,188 (20.9) | 1,149 (24.9) | **< 0.001** |
| Q2 (1,462.0-1,971.0) | 5,384 (24.7) | 787 (22.0) | 1,120 (24.7) | 2,401 (24.9) | 1,076 (25.2) |  |
| Q3 (1,971.0-2,632.5) | 5,384 (26.2) | 821 (26.2) | 1,023 (23.3) | 2,479 (26.3) | 1,061 (27.8) |  |
| Q4 (> 2,632.5) | 5,386 (27.2) | 871 (30.1) | 1,085 (25.6) | 2,605 (27.9) | 825 (22.1) |  |
| **Survey cycle** |  |  |  |  |  |  |
| 2005-2006 | 2,876 (14.7) | 559 (13.8) | 636 (14.4) | 1,478 (15.5) | 203 (10.8) | 0.728 |
| 2007-2008 | 3,353 (13.8) | 566 (13.9) | 635 (13.5) | 1,690 (14.3) | 462 (10.7) |  |
| 2009-2010 | 3,479 (13.9) | 610 (13.9) | 585 (13.8) | 1,799 (14.2) | 485 (11.9) |  |
| 2011-2012 | 3,064 (14.6) | 293 (13.5) | 792 (15.0) | 1,229 (14.6) | 750 (15.4) |  |
| 2013-2014 | 3,381 (15.3) | 439 (17.0) | 673 (16.0) | 1,520 (14.8) | 749 (16.9) |  |
| 2015-2016 | 3,053 (14.8) | 521 (14.9) | 617 (14.4) | 1,108 (14.5) | 807 (17.0) |  |
| 2017-2018 | 2,337 (12.8) | 300 (13.0) | 533 (12.9) | 849 (12.0) | 655 (17.3) |  |
| **CVH level** |  |  |  |  |  |  |
| Low | 2,634 (9.7) | 1,156 (9.3) | 772 (15.4) | 349 (8.8) | 357 (8.1) | **< 0.001** |
| Moderate | 14,508 (66.2) | 6,425 (65.6) | 3,154 (71.0) | 2,327 (69.2) | 2,602 (63.2) |  |
| High | 4,401 (24.1) | 2,092 (25.1) | 545 (13.7) | 612 (21.9) | 1,152 (28.7) |  |
| **LE8 metric scores** | | | | | |  |
| Total | 68.58 ± 0.25 | 68.15 ± 0.38 | 63.96 ± 0.30 | 68.97 ± 0.31 | 70.50 ± 0.40 | **< 0.001** |
| Diet | 38.75 ± 0.50 | 36.79 ± 0.85 | 33.48 ± 0.75 | 39.06 ± 0.62 | 42.71 ± 0.68 | **< 0.001** |
| Physical activity | 72.95 ± 0.50 | 67.90 ± 1.26 | 66.87 ± 1.00 | 74.62 ± 0.59 | 71.27 ± 0.94 | **< 0.001** |
| Nicotine exposure | 70.98 ± 0.53 | 76.67 ± 1.00 | 69.19 ± 0.87 | 69.91 ± 0.73 | 75.30 ± 0.94 | **< 0.001** |
| Sleep health | 83.64 ± 0.29 | 83.63 ± 0.56 | 73.71 ± 0.54 | 85.19 ± 0.35 | 82.73 ± 0.51 | **< 0.001** |
| Body mass index | 60.15 ± 0.44 | 54.55 ± 0.83 | 52.09 ± 0.61 | 61.00 ± 0.51 | 65.63 ± 0.85 | **< 0.001** |
| Blood lipids | 64.36 ± 0.35 | 64.08 ± 0.69 | 71.02 ± 0.49 | 63.25 ± 0.44 | 65.59 ± 0.73 | **< 0.001** |
| Blood glucose | 86.82 ± 0.25 | 85.00 ± 0.69 | 80.81 ± 0.46 | 88.06 ± 0.30 | 85.62 ± 0.45 | **< 0.001** |
| Blood pressure | 71.02 ± 0.34 | 76.58 ± 0.64 | 64.53 ± 0.58 | 70.66 ± 0.42 | 75.13 ± 0.62 | **< 0.001** |
| Footnotes: Continuous variables are presented as mean ± SE, and categorical variables are presented as n (weighted %).  ^a^ The *P*-values were assessed by One-way ANOVA (continuous variables) or by Rao-Scott chi-square test (categorical variables). *P*-values presented with bold values were statistically significant.  Abbreviations: AA, Associate's Degree; CVD, Cardiovascular disease; GED, General educational development; DM, Diabetes mellitus; IFG, Impaired fasting glycaemia; IGT, Impaired glucose tolerance; LE8, Life’s Essential 8; MetS, Metabolic syndrome; NHANES, National Health and Nutrition Examination Survey; PIR, Poverty income ratio; Q, Quartile; SE, Standard error. | | | | | | |

| **Table S5 Survey-weighted characteristic of the study population by MetS status, NHANES 2005-2018 (n = 21,543).** | | | | |
| --- | --- | --- | --- | --- |
| **Characteristics** | **Total** | **MetS status** | | ***P*-value^a^** |
|  |  | **Yes** | **No** |  |
| **Age (years)** | 46.38 ± 0.25 | 52.68 ± 0.26 | 43.51 ± 0.28 | **< 0.001** |
| 20-39 | 7,534 (36.3) | 1,324 (19.7) | 6,210 (43.9) | **< 0.001** |
| 40-59 | 7,704 (40.5) | 2,794 (45.2) | 4,910 (38.3) |  |
| 60-79 | 6,305 (23.2) | 3,097 (35.1) | 3,208 (17.7) |  |
| **Gender** |  |  |  |  |
| Female | 10,961 (51.2) | 3,855 (51.8) | 7,106 (50.9) | 0.409 |
| Male | 10,582 (48.8) | 3,360 (48.2) | 7,222 (49.1) |  |
| **Race/ethnicity** |  |  |  |  |
| Non-Hispanic White | 9,673 (70.8) | 3,431 (73.4) | 6,242 (69.6) | **< 0.001** |
| Non-Hispanic Black | 4,471 (10.0) | 1,338 (8.6) | 3,133 (10.6) |  |
| Mexican American | 3,288 (7.7) | 1,246 (8.2) | 2,042 (7.4) |  |
| Other races | 4,111 (11.5) | 1,200 (9.7) | 2,911 (12.4) |  |
| **Education level** |  |  |  |  |
| Less than 9th grade | 1,743 (3.9) | 762 (5.1) | 981 (3.4) | **< 0.001** |
| 9-11th grade (including 12th grade with no diploma) | 2,842 (9.5) | 1,074 (10.9) | 1,768 (8.8) |  |
| High school graduate/GED or equivalent | 4,896 (22.6) | 1,857 (27.4) | 3,039 (20.5) |  |
| Some college or AA degree | 6,621 (32.1) | 2,209 (33.3) | 4,412 (31.5) |  |
| College graduate or above | 5,441 (31.9) | 1,313 (23.4) | 4,128 (35.8) |  |
| **PIR** | 3.13 ± 0.04 | 3.00 ± 0.04 | 3.18 ± 0.04 | **< 0.001** |
| < 1.3 | 6,363 (19.1) | 2,363 (20.6) | 4,000 (18.3) | **< 0.001** |
| 1.3-3.5 | 8,003 (34.8) | 2,747 (37.1) | 5,256 (33.8) |  |
| > 3.5 | 7,177 (46.1) | 2,105 (42.2) | 5,072 (47.9) |  |
| **Marital status** |  |  |  |  |
| Never married | 3,972 (17.5) | 760 (10.1) | 3,212 (20.9) | **< 0.001** |
| Widowed/Divorced/Separated | 4,303 (16.6) | 1,869 (21.4) | 2,434 (14.5) |  |
| Married/Living with partner | 13,268 (65.8) | 4,586 (68.5) | 8,682 (64.6) |  |
| **Alcohol consumption** |  |  |  |  |
| Current | 15,473 (77.5) | 4,610 (70.5) | 10,863 (80.6) | **< 0.001** |
| Former | 3,396 (13.0) | 1,593 (18.7) | 1,803 (10.3) |  |
| Never | 2,674 (9.6) | 1,012 (10.7) | 1,662 (9.1) |  |
| **Hypertension** |  |  |  |  |
| Yes | 8,650 (35.9) | 4,822 (64.1) | 3,828 (23.1) | **< 0.001** |
| No | 12,893 (64.1) | 2,393 (35.9) | 10,500 (76.9) |  |
| **CVD** |  |  |  |  |
| Yes | 1,960 (7.2) | 1,215 (14.3) | 745 (3.9) | **< 0.001** |
| No | 19,583 (92.8) | 6,000 (85.7) | 13,583 (96.1) |  |
| **DM** |  |  |  |  |
| DM | 3,360 (11.7) | 2,639 (30.2) | 721 (3.4) | **< 0.001** |
| IFG | 986 (4.7) | 597 (9.6) | 389 (2.4) |  |
| IGT | 871 (3.6) | 357 (4.9) | 514 (3.1) |  |
| No | 16,326 (79.9) | 3,622 (55.3) | 12,704 (91.2) |  |
| **Total energy intake (kcal)** | 2,208.88 ± 9.56 | 2,152.11 ± 16.57 | 2,234.68 ± 11.47 | **< 0.001** |
| Q1 (< 1,462.0) | 5,389 (21.9) | 2,000 (22.8) | 3,389 (21.5) | **< 0.05** |
| Q2 (1,462.0-1,971.0) | 5,384 (24.7) | 1,874 (25.8) | 3,510 (24.2) |  |
| Q3 (1,971.0-2,632.5) | 5,384 (26.2) | 1,778 (26.4) | 3,606 (26.1) |  |
| Q4 (> 2,632.5) | 5,386 (27.2) | 1,563 (25.0) | 3,823 (28.2) |  |
| **Survey cycle** |  |  |  |  |
| 2005-2006 | 2,876 (14.7) | 830 (13.1) | 2,046 (15.5) | **< 0.05** |
| 2007-2008 | 3,353 (13.8) | 1,223 (14.6) | 2,130 (13.4) |  |
| 2009-2010 | 3,479 (13.9) | 1,138 (13.0) | 2,341 (14.3) |  |
| 2011-2012 | 3,064 (14.6) | 950 (14.0) | 2,114 (14.9) |  |
| 2013-2014 | 3,381 (15.3) | 1,131 (15.5) | 2,250 (15.3) |  |
| 2015-2016 | 3,053 (14.8) | 1,149 (17.0) | 1,904 (13.9) |  |
| 2017-2018 | 2,337 (12.8) | 794 (12.8) | 1,543 (12.8) |  |
| **LE8 metric scores** | | | |  |
| Total | 68.58 ± 0.25 | 58.87 ± 0.24 | 73.00 ± 0.24 | **< 0.001** |
| Diet | 38.75 ± 0.50 | 36.15 ± 0.59 | 39.93 ± 0.57 | **< 0.001** |
| Physical activity | 72.95 ± 0.50 | 66.05 ± 0.77 | 76.08 ± 0.53 | **< 0.001** |
| Nicotine exposure | 70.98 ± 0.53 | 69.99 ± 0.61 | 71.42 ± 0.63 | **< 0.05** |
| Sleep health | 83.64 ± 0.29 | 82.42 ± 0.42 | 84.19 ± 0.32 | **< 0.001** |
| Body mass index | 60.15 ± 0.44 | 37.23 ± 0.48 | 70.57 ± 0.41 | **< 0.001** |
| Blood lipids | 64.36 ± 0.35 | 52.04 ± 0.50 | 69.96 ± 0.38 | **< 0.001** |
| Blood glucose | 86.82 ± 0.25 | 72.14 ± 0.53 | 93.49 ± 0.19 | **< 0.001** |
| Blood pressure | 71.02 ± 0.34 | 54.91 ± 0.46 | 78.34 ± 0.34 | **< 0.001** |
| Footnotes: Continuous variables are presented as mean ± SE, and categorical variables are presented as n (weighted %).  ^a^ The *P*-values were assessed by *T*-test (continuous variables) or by Rao-Scott chi-square test (categorical variables). *P*-values presented with bold values were statistically significant.  Abbreviations: AA, Associate's Degree; CVD, Cardiovascular disease; GED, General educational development; DM, Diabetes mellitus; IFG, Impaired fasting glycaemia; IGT, Impaired glucose tolerance; LE8, Life’s Essential 8; MetS, Metabolic syndrome; NHANES, National Health and Nutrition Examination Survey; PIR, Poverty income ratio; Q, Quartile; SE, Standard error. | | | | |

| **Table S6 Association of LE8 scores with the risk of MetS in the female population, NHANES 2005-2018 (n = 10,961).** | | | | | | | | | |
| --- | --- | --- | --- | --- | --- | --- | --- | --- | --- |
|  | **Cases/Participants** | **Crude model** | |  | **Model 1** | |  | **Model 2** | |
|  |  | **COR (95% CI)** | ***P-*value** |  | **AOR (95% CI)** | ***P*-value** |  | **AOR (95% CI)** | ***P*-value** |
| **Total** |  |  |  |  |  |  |  |  |  |
| Low (0-49) | 997/1,351 | Reference | - |  | Reference | - |  | Reference | - |
| Moderate (50-79) | 2,697/6,957 | **0.200 (0.172, 0.233)** | **< 0.001** |  | **0.208 (0.178, 0.242)** | **< 0.001** |  | **0.224 (0.191, 0.262)** | **< 0.001** |
| High (80-100) | 161/2,653 | **0.016 (0.013, 0.020)** | **< 0.001** |  | **0.019 (0.015, 0.024)** | **< 0.001** |  | **0.023 (0.018, 0.029)** | **< 0.001** |
| *P* for trend |  |  | **< 0.001** |  |  | **< 0.001** |  |  | **< 0.001** |
| **Diet** |  |  |  |  |  |  |  |  |  |
| Low (0-49) | 1,932/5,204 | Reference | - |  | Reference | - |  | Reference | - |
| Moderate (50-79) | 966/2,747 | 0.861 (0.740, 1.002) | 0.054 |  | **0.721 (0.616, 0.843)** | **< 0.001** |  | 0.948 (0.774, 1.162) | 0.604 |
| High (80-100) | 957/3,010 | **0.652 (0.566, 0.752)** | **< 0.001** |  | **0.482 (0.417, 0.558)** | **< 0.001** |  | 0.842 (0.689, 1.029) | 0.091 |
| *P* for trend |  |  | **< 0.001** |  |  | **< 0.001** |  |  | 0.109 |
| **Physical activity** |  |  |  |  |  |  |  |  |  |
| Low (0-49) | 1,593/3,746 | Reference | - |  | Reference | - |  | Reference | - |
| Moderate (50-79) | 208/588 | **0.720 (0.580, 0.895)** | **< 0.05** |  | **0.788 (0.622, 0.999)** | **< 0.05** |  | 0.837 (0.625, 1.121) | 0.229 |
| High (80-100) | 2,054/6,627 | **0.563 (0.500, 0.634)** | **< 0.001** |  | **0.649 (0.570, 0.740)** | **< 0.001** |  | 0.976 (0.811, 1.176) | 0.799 |
| *P* for trend |  |  | **< 0.001** |  |  | **< 0.001** |  |  | 0.858 |
| **Nicotine exposure** |  |  |  |  |  |  |  |  |  |
| Low (0-49) | 859/2,169 | Reference | - |  | Reference | - |  | Reference | - |
| Moderate (50-79) | 770/1,877 | 0.928 (0.797, 1.082) | 0.338 |  | **0.625 (0.525, 0.745)** | **< 0.001** |  | **0.564 (0.446, 0.712)** | **< 0.001** |
| High (80-100) | 2,215/6,895 | **0.678 (0.603, 0.762)** | **< 0.001** |  | **0.582 (0.510, 0.663)** | **< 0.001** |  | **0.568 (0.468, 0.688)** | **< 0.001** |
| *P* for trend |  |  | **< 0.001** |  |  | **< 0.001** |  |  | **< 0.001** |
| **Sleep health** |  |  |  |  |  |  |  |  |  |
| Low (0-49) | 743/1,898 | Reference | - |  | Reference | - |  | Reference | - |
| Moderate (50-79) | 789/2,239 | **0.787 (0.661, 0.937)** | **< 0.05** |  | **0.759 (0.632, 0.911)** | **< 0.001** |  | 1.038 (0.827, 1.303) | 0.745 |
| High (80-100) | 2,323/2,824 | **0.752 (0.645, 0.876)** | **< 0.001** |  | **0.705 (0.600, 0.828)** | **< 0.001** |  | 1.128 (0.930, 1.368) | 0.218 |
| *P* for trend |  |  | **< 0.001** |  |  | **< 0.001** |  |  | 0.171 |
| **Body mass index** |  |  |  |  |  |  |  |  |  |
| Low (0-49) | 2,523/4,629 | Reference | - |  | Reference | - |  | Reference | - |
| Moderate (50-79) | 1,037/3,045 | **0.365 (0.321, 0.415)** | **< 0.001** |  | **0.320 (0.278, 0.369)** | **< 0.001** |  | **0.389 (0.336, 0.450)** | **< 0.001** |
| High (80-100) | 295/3,287 | **0.061 (0.051, 0.073)** | **< 0.001** |  | **0.058 (0.048, 0.069)** | **< 0.001** |  | **0.080 (0.066, 0.097)** | **< 0.001** |
| *P* for trend |  |  | **< 0.001** |  |  | **< 0.001** |  |  | **< 0.001** |
| **Blood lipids** |  |  |  |  |  |  |  |  |  |
| Low (0-49) | 1,184/3,434 | Reference | - |  | Reference | - |  | Reference | - |
| Moderate (50-79) | 779/2,601 | **0.347 (0.300, 0.402)** | **< 0.001** |  | **0.409 (0.349, 0.480)** | **< 0.001** |  | **0.478 (0.399, 0.574)** | **< 0.001** |
| High (80-100) | 1,192/4,926 | **0.237 (0.210, 0.268)** | **< 0.001** |  | **0.296 (0.259, 0.337)** | **< 0.001** |  | **0.349 (0.299, 0.406)** | **< 0.001** |
| *P* for trend |  |  | **< 0.001** |  |  | **< 0.001** |  |  | **< 0.001** |
| **Blood glucose** |  |  |  |  |  |  |  |  |  |
| Low (0-49) | 1,109/1,300 | Reference | - |  | Reference | - |  | Reference | - |
| Moderate (50-79) | 1,167/2,107 | **0.218 (0.167, 0.283)** | **< 0.001** |  | **0.222 (0.171, 0.289)** | **< 0.001** |  | **0.226 (0.168, 0.304)** | **< 0.001** |
| High (80-100) | 1,579/7,554 | **0.043 (0.033, 0.056)** | **< 0.001** |  | **0.052 (0.040, 0.068)** | **< 0.001** |  | **0.073 (0.054, 0.098)** | **< 0.001** |
| *P* for trend |  |  | **< 0.001** |  |  | **< 0.001** |  |  | **< 0.001** |
| **Blood pressure** |  |  |  |  |  |  |  |  |  |
| Low (0-49) | 1,005/1,665 | Reference | - |  | Reference | - |  | Reference | - |
| Moderate (50-79) | 1,160/2,992 | **0.375 (0.315, 0.445)** | **< 0.001** |  | **0.443 (0.371, 0.529)** | **< 0.001** |  | **0.427 (0.342, 0.533)** | **< 0.001** |
| High (80-100) | 1,310/5,752 | **0.179 (0.153, 0.209)** | **< 0.001** |  | **0.260 (0.220, 0.307)** | **< 0.001** |  | **0.345 (0.276, 0.429)** | **< 0.001** |
| *P* for trend |  |  | **< 0.001** |  |  | **< 0.001** |  |  | **< 0.001** |
| Footnotes: For the total LE8 score: The crude model was unadjusted. Model 1 was adjusted for age, gender, and race/ethnicity. Model 2 was adjusted for age, gender, race/ethnicity, education level, marital status, PIR, and alcohol consumption. For the 8 LE8 metrics scores: Model 2 adjusted for gender, age, race/ethnicity, education level, marital status, PIR, alcohol consumption, diet, nicotine exposure, physical activity, sleep health, body mass index, blood glucose, blood lipids, and blood pressure. When the association between each LE8 metric and MetS was evaluated, this metric was excluded from the adjustment. The results of COR (95% CI), AOR (95% CI), and P-value shown in bold were statistically significant. *P*-value < 0.05 or *P*-value < 0.001.  Abbreviations: AOR, Adjusted odds ratio; CI, Confidence interval; COR, Crude odds ratio; LE8, Life’s Essential 8; MetS, Metabolic syndrome; NHANES, National Health and Nutrition Examination Survey; PIR, Poverty income ratio. | | | | | | | | | |

| **Table S7 Association of LE8 scores with the risk of MetS in the male population, NHANES 2005-2018 (n = 10,582).** | | | | | | | | | |
| --- | --- | --- | --- | --- | --- | --- | --- | --- | --- |
|  | **Cases/Participants** | **Crude model** | |  | **Model 1** | |  | **Model 2** | |
|  |  | **COR (95% CI)** | ***P-*value** |  | **AOR (95% CI)** | ***P*-value** |  | **AOR (95% CI)** | ***P*-value** |
| **Total** |  |  |  |  |  |  |  |  |  |
| Low (0-49) | 865/1,283 | Reference | - |  | Reference | - |  | Reference | - |
| Moderate (50-79) | 2,402/7,551 | **0.241 (0.204, 0.284)** | **< 0.001** |  | **0.253 (0.214, 0.301)** | **< 0.001** |  | **0.243 (0.202, 0.292)** | **< 0.001** |
| High (80-100) | 93/1,748 | **0.028 (0.020, 0.040)** | **< 0.001** |  | **0.032 (0.022, 0.045)** | **< 0.001** |  | **0.030 (0.021, 0.044)** | **< 0.001** |
| *P* for trend |  |  | **< 0.001** |  |  | **< 0.001** |  |  | **< 0.001** |
| **Diet** |  |  |  |  |  |  |  |  |  |
| Low (0-49) | 1,852/5,782 | Reference | - |  | Reference | - |  | Reference | - |
| Moderate (50-79) | 824/2,584 | 0.944 (0.830, 1.074) | 0.377 |  | **0.808 (0.702, 0.929)** | **< 0.05** |  | 0.959 (0.789, 1.166) | 0.671 |
| High (80-100) | 684/2,216 | 0.858 (0.727, 1.012) | 0.069 |  | **0.631 (0.529, 0.752)** | **< 0.001** |  | 0.903 (0.731, 1.117) | 0.342 |
| *P* for trend |  |  | 0.069 |  |  | **< 0.001** |  |  | 0.338 |
| **Physical activity** |  |  |  |  |  |  |  |  |  |
| Low (0-49) | 1,035/2,682 | Reference | - |  | Reference | - |  | Reference | - |
| Moderate (50-79) | 148/445 | 0.903 (0.702, 1.162) | 0.423 |  | 0.995 (0.770, 1.285) | 0.968 |  | 1.401 (0.976, 2.013) | 0.067 |
| High (80-100) | 2,177/7,455 | **0.682 (0.596, 0.780)** | **< 0.001** |  | **0.786 (0.683, 0.903)** | **< 0.001** |  | 0.992 (0.836, 1.177) | 0.928 |
| *P* for trend |  |  | **< 0.001** |  |  | **< 0.001** |  |  | 0.701 |
| **Nicotine exposure** |  |  |  |  |  |  |  |  |  |
| Low (0-49) | 765/2,867 | Reference | - |  | Reference | - |  | Reference | - |
| Moderate (50-79) | 1,169/2,726 | **2.086 (1.750, 2.486)** | **< 0.001** |  | **1.398 (1.156, 1.689)** | **< 0.001** |  | 1.108 (0.864, 1.419) | 0.414 |
| High (80-100) | 1,415/4,952 | **1.198 (1.034, 1.388)** | **< 0.05** |  | 1.160 (0.998, 1.348) | 0.053 |  | 1.041 (0.849, 1.276) | 0.699 |
| *P* for trend |  |  | 0.496 |  |  | 0.203 |  |  | 0.832 |
| **Sleep health** |  |  |  |  |  |  |  |  |  |
| Low (0-49) | 605/1,808 | Reference | - |  | Reference | - |  | Reference | - |
| Moderate (50-79) | 795/2,460 | 0.860 (0.717, 1.032) | 0.104 |  | 0.821 (0.670, 1.006) | 0.058 |  | 0.892 (0.681, 1.168) | 0.401 |
| High (80-100) | 1,958/6,314 | 0.873 (0.753, 1.012) | 0.071 |  | **0.792 (0.675, 0.930)** | **< 0.05** |  | 0.887 (0.723, 1.088) | 0.246 |
| *P* for trend |  |  | 0.149 |  |  | **< 0.05** |  |  | 0.294 |
| **Body mass index** |  |  |  |  |  |  |  |  |  |
| Low (0-49) | 2,256/3,809 | Reference | - |  | Reference | - |  | Reference | - |
| Moderate (50-79) | 965/4,000 | **0.200 (0.174, 0.230)** | **< 0.001** |  | **0.172 (0.148, 0.200)** | **< 0.001** |  | **0.194 (0.164, 0.229)** | **< 0.001** |
| High (80-100) | 139/2,773 | **0.026 (0.020, 0.036)** | **< 0.001** |  | **0.025 (0.019, 0.034)** | **< 0.001** |  | **0.030 (0.022, 0.042)** | **< 0.001** |
| *P* for trend |  |  | **< 0.001** |  |  | **< 0.001** |  |  | **< 0.001** |
| **Blood lipids** |  |  |  |  |  |  |  |  |  |
| Low (0-49) | 1,621/3,842 | Reference | - |  | Reference | - |  | Reference | - |
| Moderate (50-79) | 596/2,488 | **0.448 (0.380, 0.527)** | **< 0.001** |  | **0.507 (0.425, 0.606)** | **< 0.001** |  | **0.608 (0.499, 0.739)** | **< 0.001** |
| High (80-100) | 1,143/4,252 | **0.478 (0.419, 0.545)** | **< 0.001** |  | **0.493 (0.436, 0.558)** | **< 0.001** |  | **0.512 (0.442, 0.593)** | **< 0.001** |
| *P* for trend |  |  | **< 0.001** |  |  | **< 0.001** |  |  | **< 0.001** |
| **Blood glucose** |  |  |  |  |  |  |  |  |  |
| Low (0-49) | 1,075/1,419 | Reference | - |  | Reference | - |  | Reference | - |
| Moderate (50-79) | 919/2,234 | **0.237 (0.187, 0.301)** | **< 0.001** |  | **0.252 (0.198, 0.319)** | **< 0.001** |  | **0.191 (0.145, 0.253)** | **< 0.001** |
| High (80-100) | 1,366/6,929 | **0.079 (0.063, 0.098)** | **< 0.001** |  | **0.099 (0.078, 0.125)** | **< 0.001** |  | **0.101 (0.077, 0.132)** | **< 0.001** |
| *P* for trend |  |  | **< 0.001** |  |  | **< 0.001** |  |  | **< 0.001** |
| **Blood pressure** |  |  |  |  |  |  |  |  |  |
| Low (0-49) | 977/1,794 | Reference | - |  | Reference | - |  | Reference | - |
| Moderate (50-79) | 1,172/4,124 | **0.291 (0.242, 0.350)** | **< 0.001** |  | **0.362 (0.296, 0.441)** | **< 0.001** |  | **0.391 (0.317, 0.482)** | **< 0.001** |
| High (80-100) | 880/4,152 | **0.183 (0.154, 0.217)** | **< 0.001** |  | **0.236 (0.197, 0.283)** | **< 0.001** |  | **0.306 (0.251, 0.372)** | **< 0.001** |
| *P* for trend |  |  | **< 0.001** |  |  | **< 0.001** |  |  | **< 0.001** |
| Footnotes: For the total LE8 score: The crude model was unadjusted. Model 1 was adjusted for age, gender, and race/ethnicity. Model 2 was adjusted for age, gender, race/ethnicity, education level, marital status, PIR, and alcohol consumption. For the 8 LE8 metrics scores: Model 2 adjusted for gender, age, race/ethnicity, education level, marital status, PIR, alcohol consumption, diet, nicotine exposure, physical activity, sleep health, body mass index, blood glucose, blood lipids, and blood pressure. When the association between each LE8 metric and MetS was evaluated, this metric was excluded from the adjustment. The results of COR (95% CI), AOR (95% CI), and *P*-value shown in bold were statistically significant. *P*-value < 0.05 or *P*-value < 0.001.  Abbreviations: AOR, Adjusted odds ratio; CI, Confidence interval; COR, Crude odds ratio; LE8, Life’s Essential 8; MetS, Metabolic syndrome; NHANES, National Health and Nutrition Examination Survey; PIR, Poverty income ratio. | | | | | | | | | |

| **Table S8 Association of LE8 score with the risk of MetS for additional adjustments, NHANES 2005-2018 (n = 21,543).** | | | | | | | | | |
| --- | --- | --- | --- | --- | --- | --- | --- | --- | --- |
|  | **LE8 score** | | | | | | | | |
|  | **Low CVH (0-49)** |  | **Moderate CVH (50-79)** | |  | **High CVH (80-100)** | |  | ***P* for trend** |
|  |  |  | **AOR (95% CI)** | ***P*-value** |  | **AOR (95% CI)** | ***P*-value** |  |  |
| **Original** | Reference |  | **0.234 (0.209, 0.262)** | **< 0.001** |  | **0.026 (0.021, 0.032)** | **< 0.001** |  | **< 0.001** |
| **Adding** |  |  |  |  |  |  |  |  |  |
| **Survey cycle** | Reference |  | **0.232 (0.207, 0.259)** | **< 0.001** |  | **0.025 (0.020, 0.031)** | **< 0.001** |  | **< 0.001** |
| **DM** | Reference |  | **0.306 (0.270, 0.347)** | **< 0.001** |  | **0.040 (0.031, 0.050)** | **< 0.001** |  | **< 0.001** |
| **Hypertension** | Reference |  | **0.283 (0.251, 0.320)** | **< 0.001** |  | **0.040 (0.032, 0.050)** | **< 0.001** |  | **< 0.001** |
| **CVD** | Reference |  | **0.243 (0.217, 0.272)** | **< 0.001** |  | **0.027 (0.021, 0.033)** | **< 0.001** |  | **< 0.001** |
| **Total energy intake** | Reference |  | **0.234 (0.209, 0.262)** | **< 0.001** |  | **0.026 (0.021, 0.032)** | **< 0.001** |  | **< 0.001** |
| **Depression** | Reference |  | **0.238 (0.212, 0.267)** | **< 0.001** |  | **0.026 (0.021, 0.033)** | **< 0.001** |  | **< 0.001** |
| Footnotes: The multivariable logistic regression model was adjusted for age, gender, race/ethnicity, education level, marital status, PIR, and alcohol consumption. Survey cycle, DM (yes or no), hypertension (yes or no), CVD (yes or no), total energy intake (kcal), and depression (yes or no) were added into the multivariable logistic regression model for additional adjustment, respectively. Results of AOR (95% CI), *P* for tend, and *P*-value presented with bold values were statistically significant with *P*-value < 0.05 or *P*-value < 0.001.  Abbreviations: AOR, Adjusted odds ratio; CI, Confidence interval; CVD, Cardiovascular disease; CVH, Cardiovascular health; DM, Diabetes mellitus; LE8, Life’s Essential 8; MetS, Metabolic syndrome; NHANES, National Health and Nutrition Examination Survey; PIR, Poverty income ratio. | | | | | | | | | |

| **Table S9 Association of LE8 score with the risk of MetS, High CVH (80-100) as the reference, NHANES 2005–2018 (n = 21,543).** | | | | | | | | |
| --- | --- | --- | --- | --- | --- | --- | --- | --- |
| **LE8 score** | **Crude model** | |  | **Model 1** | |  | **Model 2** | |
|  | **COR (95% CI)** | ***P-*value** |  | **AOR (95% CI)** | ***P*-value** |  | **AOR (95% CI)** | ***P*-value** |
| High CVH (80-100) | Reference | - |  | Reference | - |  | Reference | - |
| Moderate CVH (50-79) | **10.363 (8.585, 12.509)** | **< 0.001** |  | **9.520 (7.867,11.519)** | **< 0.001** |  | **9.117 (7.512,11.064)** | **< 0.001** |
| Low CVH (0-49) | **47.380 (38.214, 58.745)** | **< 0.001** |  | **41.183 (33.276,50.969)** | **< 0.001** |  | **38.960 (31.208,48.637)** | **< 0.001** |
| *P* for trend |  | **< 0.001** |  |  | **< 0.001** |  |  | **< 0.001** |
| Footnotes: The crude model was unadjusted. Model 1 was adjusted for age, gender, and race/ethnicity. Model 2 was adjusted for age, gender, race/ethnicity, education level, marital status, PIR, and alcohol consumption. Results of COR (95% CI), AOR (95% CI), *P* for tend, and *P*-value presented with bold values were statistically significant with *P*-value < 0.05 or *P*-value < 0.001.  Abbreviations: AOR, Adjusted odds ratio; CI, Confidence interval; COR, Crude odds ratio; CVH, Cardiovascular health; LE8, Life’s Essential 8; MetS, Metabolic syndrome; NHANES, National Health and Nutrition Examination Survey; PIR, Poverty income ratio. | | | | | | | | |
